# The Work of the Metropolitan Asylums Board

**Published:** 1917-08-18

**Authors:** 


### THE GUARDIAN OF LONDON'S HEALTH.

### The Work of the Metropolitan Asylums Board.

The social and sanitary problem of the large city is a very complex one, and it has reached its highest expression in our own Metropolis. Fortunately, its development has moved gradually rather than by leaps, and in this way the solution demanded has had time for growth. Not everything which wisdom after the event could have desired has been foreseen. Yet there has been foresight as well as achievement; and, tested on the practical plane, the municipal history of London is not unworthy of the fame of the city. On the æsthetic and artistic side there is doubtless room for criticism, but the essential needs of a vast population have been met with a large measure of success. The future may bring higher standards, and with them may come condemnation. The fair test, however, is the capacity and opportunity of the contemporary hour, and if here there is justification posterity may be trusted for a reasonable judgment. Tried by existing possibilities, and not by those which may be available in days to come, the issue may reasonably count on a liberal verdict, though there will be qualifications in the decision expressed by the tribunal of the next generation. Here and now it is much to be able to speak with satisfaction of the agencies which stand for the protection of health against the multiplied dangers involved in a community of some seven millions of souls.

### Fever Provision Costs £422,313.

A conspicuous position among these agencies is that occupied by the organisation known as the Metropolitan Asylums Board. The title is not altogether a fortunate one, inasmuch as it suggests that the work of the Board is limited to the provision of institutions for the care of the insane, whereas, as a matter of fact, this is only one—and by no means the most important—of many and various activities. Hospitals for infectious diseases, of which there are fourteen, occupy perhaps the principal position in the scheme, but in addition are included sanatoria for consumptives, asylums and colonies for the imbecile and feeble-minded, hospitals for sick and convalescent children, schools for children suffering from ophthalmia, ambulance service, including a department concerned with the river, casual wards, a training ship, a bateriological establishment, and the administrative service which controls and directs the whole organisation. It is the staff of these institutions which stand between London and the terrors of epidemic disease, not to speak of more limited social disasters. A few figures may be quoted in illustration of this point. Thus during last year over 20,000 persons suffering from infectious disease were admitted into the Board's hospitals, and the figures were less by some 7,000 than those of 1915. The expense of this department, exclusive of any share of the head office and other central charges, was £422,313. Cases of tuberculosis numbered more than 2,500, admissions to children's hospitals, schools, and homes reached a total of 2,506, and the figures of the casual poor received during the year approached 10,000. These are instances taken at haphazard, but they are sufficient to show with how large a population the Board's activities are immediately concerned and from what mischiefs these activities protect the community. The financial responsibilities show necessarily a corresponding measure. The general expenditure of the year was over £1,200,000, and would have been more than this but for the fact that the War Office has taken over four of the Board's hospitals.

### Its Central Authority and Extensions.

A point worthy of note is the gradual extension of function which time has brought to the Board. Constituted in 1867 by an Order of the Poor-Law Board, its immediate purpose was to establish "asylums" for the reception and relief of the sick, insane, or infirm. Gradually it has taken over all the Poor-Law work of the Metropolis which can be best transacted by a central authority rather than by the local Boards of Guardians. Its extension in other directions is indicated by the list of the various institutions guided by its control and roughly indicated in a preceding paragraph. Nor has its capacity for growth become exhausted. Even in 1916 the Local Government Board imposed on it two new orders of constituents—namely, parturient women suffering from venereal disease, and sane epileptic children—while in the current year adult sane epileptics were added. The family is indeed a large and varied one, and to provide for its wants there must be a correspondingly elaborate organisation.

### A Lamentable Ignorance.

Yet the citizens of London, speaking generally, know little about the Metropolitan Asylums Board. Even members of the medical profession often show themselves to be but slightly acquainted with its constitution and activities apart from the reception and treatment of cases of infectious disease. One reason for this doubtless is that the Board and its officials eschew the gentle art of self-advertisement. Beyond a series of statistical announcements, which do not readily engage popular attention, the Board's activities make but rare appearance in newspaper columns, and the man in the street knows little or nothing of them. Yet, in addition to the salvation which they bring to many of the sick and suffering, they stand as protecting agencies around each one of us. It is a privilege to utilise this brief opportunity to turn the thoughts of our readers to an organisation which quietly effects such beneficent ends. We have known the work of the Board and its wise and steady developments for over forty years. Residents of London could not spend any leisure they command better or more interestingly than by studying the work and examining the organisations of the Asylums Board. They would find it an education in many fields of work, and an inspiring and helpful experience in every way.

